# Bioactive Food Proteins: Bridging Nutritional and Functional Benefits with Sustainable Protein Sources

**DOI:** 10.3390/foods14173035

**Published:** 2025-08-29

**Authors:** Manuela Machado, Israel Bautista-Hérnandez, Ricardo Gómez-García, Sara Silva, Eduardo M. Costa

**Affiliations:** CBQF Centro de Biotecnologia e Química Fina-Laboratório Associado, Escola Superior de Biotecnologia, Universidade Católica Portuguesa, Rua Diogo Botelho 1327, 4169-005 Porto, Portugal; mmachado@ucp.pt (M.M.); s-ibhernandez@ucp.pt (I.B.-H.); rgarcia@ucp.pt (R.G.-G.); snsilva@ucp.pt (S.S.)

**Keywords:** bioactive peptides, alternative proteins, fermentation, sustainable nutrition, functional food

## Abstract

Bioactive food proteins play multifunctional roles in human health and functional food development. Beyond their nutritional value, these proteins contain peptide sequences capable of exerting physiological effects, such as antioxidant, anti-hypertensive, immunomodulatory, and anti-inflammatory activities. This review summarises the processing and functional technologies applied to bioactive proteins; the increasing use of alternative protein sources including plants, microorganisms, and insects; and how these proteins exert their activity. Advances in high-tech production methods—such as fermentation and cultured meat—are also discussed, alongside current challenges related to safety, regulation, and consumer acceptance. Bibliometric and patent analyses further demonstrate sustained innovation and interest in this field, highlighting the potential of bioactive proteins to contribute to sustainable, health-promoting food systems.

## 1. Introduction

The global food system is undergoing a profound transformation driven by population growth, climate change, and growing awareness of health and sustainability. By 2050, the world population is expected to reach 9.7 billion, requiring a substantial increase in food production while simultaneously reducing environmental impact. Proteins, as essential macronutrients, are at the heart of this challenge: they are indispensable for human growth and health, yet conventional production methods are associated with high ecological and ethical costs [1]. Livestock farming alone contributes nearly 14.5% of global greenhouse gas emissions and requires disproportionate land and water resources, raising concerns about the long-term sustainability of animal protein [2].

At the same time, the role of proteins is being redefined. Once considered primarily as sources of amino acids and energy, they are now recognised as reservoirs of bioactive peptides capable of modulating physiological processes. Such peptides have demonstrated antihypertensive, antioxidant, immunomodulatory, anti-inflammatory, and metabolic effects, suggesting applications not only in basic nutrition but also in the prevention and management of chronic diseases [3]. This dual perspective—nutritional and functional—positions proteins at the centre of both food security and health innovation [4,5,6,7].

In recent years, numerous reviews have explored aspects of bioactive proteins and peptides, including their occurrence in food, health effects, and methods of production. Other reviews have addressed the sustainability of alternative protein sources such as plants, insects, microalgae, and cultured meat. However, these two research streams—bioactivity on one side and sustainability on the other—have rarely been integrated. Most reviews tend either to focus narrowly on biochemical mechanisms and health benefits, without considering the feasibility of producing bioactive proteins at scale from sustainable sources, or to survey emerging proteins primarily from an environmental and nutritional standpoint, with limited attention to their bioactive potential and functional applications [7,8,9].

This separation leaves an important gap. There is no comprehensive synthesis that bridges novel and sustainable protein sources with the technological strategies required to unlock bioactivity and the applications that connect these proteins to consumer health and market realities. Addressing this gap is crucial, as the next generation of protein innovation must integrate sustainability with demonstrable health benefits in order to meet societal needs and regulatory expectations. The purpose of this review is therefore to provide an integrated perspective on bioactive proteins and peptides, presenting novel and alternative protein sources alongside conventional ones, examining the structural and mechanistic aspects that determine their bioactivity, evaluating technological strategies that enhance peptide release and stability, and discussing their applications in functional foods, nutraceuticals, and clinical nutrition. Finally, the review also considers the market trends, consumer acceptance, and challenges that will shape the successful integration of bioactive proteins into future food systems.

## 2. Protein Sources: From Conventional to Revolutionary

As the world’s food systems face growing challenges like population growth, climate change, and limited resources, finding new sources of protein has become more important than ever. While traditional animal and plant proteins offer good nutrition, they come with environmental and ethical concerns, especially when considering future food demands. To address the dual challenge of ensuring global nutrition and reducing environmental burden, a range of novel protein sources has emerged. These alternatives—derived from plants, insects, microbes, and cell culture—are increasingly investigated not only for their nutritional quality but also for their capacity to generate bioactive peptides with health-promoting properties.

### 2.1. Conventional Sources

While conventional animal proteins continue to provide valuable nutrition, their sustainability is limited, with the major drawbacks associated with animal-based proteins being deforestation, high greenhouse gas emissions, and intensive water and land use [10]. So, with over 9 billion people estimated by 2050, and dietary trends which favour high protein diets due to their satiety, high caloric intake and beneficial health effects chronic diseases and obesity [11,12], a clear need exists for alternative protein sources capable of meeting nutritional demands without exacerbating environmental impacts [13].

Plant-based proteins appear, naturally, as the first logical option, but despite being generally less impactful, they may also strain land and water resources if poorly managed [14]. On the other hand, microbial protein production is a more sustainable and promising alternative for high quality protein production with lower environmental impact, due to advances in process yield, efficiency and scalability [15].

### 2.2. Alternative Sources

#### 2.2.1. Plant Based Proteins

With livestock production’s lack of sustainability and high environmental impact being presented as an issue, interest in plant-based protein sources has grown [16]. Cereals, legumes, soy, and pseudocereals (e.g., amaranth, quinoa) are increasingly integrated into human diets as replacements or complements to animal protein [17]. Furthermore, plant proteins offer several advantages: cereals contain 6–15% protein, nuts 8–38%, and legumes 20–38%, with protein digestibility-corrected amino acid scores (PDCAAS) comparable to animal sources (e.g., legumes 81–96%, amaranth 90%, quinoa 73%, egg 81.4%, beef 74%) [18]. However, the low content of essential amino acids such as lysine or methionine is a limitation in plant-based diets, which can be overcome by increasing intake, combining diverse plant sources, or fortifying products with the missing amino acids [19].

From an environmental perspective, plant protein production has been shown to produce lower greenhouse gas emissions than animal proteins, although economic yields per hectare may still favour livestock [20]. With this problem in mind, over the last decade efforts have focused on advances on extraction technologies, including enzymatic methods. This has led to improvements in protein yield and amino acid quality from crops such as safflower (75% extraction, 41% essential amino acids) and sugar beet (up to 79% yield) [21,22]. Nevertheless, further research is needed to optimise plant protein integration into balanced diets.

#### 2.2.2. Insect-Based Proteins

Insects have long been part of culinary traditions in many cultures, with commonly consumed species including termites, beetles, locusts, crickets, and caterpillars [23]. Their nutritional value is notable, with protein content ranging from 40 to 75%, high levels of essential amino acids, B vitamins, unsaturated fatty acids, and minerals. Insect farming also requires less land, water, and energy and produces fewer greenhouse gas emissions compared with livestock [17,24].

Enzymatic hydrolysis of insect proteins can generate bioactive peptides with antioxidant, antimicrobial, and antihypertensive activities [25,26]. However, consumer acceptance remains a barrier in Western countries due to cultural perceptions, sensory attributes, and “disgust” factors. Processing insects into powders has helped overcome these challenges, with such products increasingly accepted in Europe and North America [27].

Applications include incorporation into meat products (e.g., mealworm- or cricket-enriched burgers), which have been shown to reduce the formation of heterocyclic amines and modulate digestive enzymes [28]. Insect powders added to soy-based burgers have enhanced nutritional profiles without compromising texture, although lipid content increased due to the fat-rich biomass [29]. Insects are also being integrated into bakery products, tortillas, and gluten-free bread [30]. Hydrolysates from species such as *Alphitobius diaperinus* have demonstrated antioxidant and ACE-inhibitory activity, highlighting their potential as functional food ingredients [31].

### 2.3. High Tech Sources

Cutting-edge biotechnologies enable the production of high-quality proteins with specific functional properties while reducing environmental burden. Microbial fermentation and cultured meat are at the forefront of this revolution, offering opportunities to valorise waste streams and design proteins for targeted health effects.

#### 2.3.1. Microbial Proteins (Single-Cell Proteins)

One of the most sought-out biotechnology tools employed in alternative protein production is fermentation-based approaches, which can be categorised into three different kinds:

1. Raw materials nutritional profile improvement (e.g., cereals, legumes).
2. Microbial biomass production with minimal processing.
3. Targeted production of specific metabolites via precision fermentation (e.g., proteins).

The microorganisms commonly employed in these processes include algae (*Spirulina platensis*, *Chlorella vulgaris*), yeasts (*Saccharomyces cerevisiae*), bacteria (*Lactobacillus* spp.), and fungi (*Aspergillus oryzae*, *Fusarium venenatum*) [32,33,34]. Examples of products with compounds produced via these methods are the mycoprotein-based sausages and burgers have shown high consumer acceptance and nutritional quality [35,36]. However, there are drawbacks, as microbial proteins may contain high levels of nucleic acids, which can increase uric acid and kidney risks. These can be mitigated via pre-treatments such as thermal or enzymatic processing [35,37].

Additionally, these processes have additional sustainability possibilities, as agro-industrial waste streams (e.g., lignocellulosic residues, dairy by-products) are promising fermentation substrates that can be used instead of complex and expensive fermentation media [34,38]. Examples of this, are the successful cultivation the yeasts *Yarrowia lipolytica* and *Candida sorboxylosa* on food waste and coffee wastewater, achieving protein contents of ca. 38% Yang, et al. [39]. However, and despite the significant cost reduction (35–75%) and waste valorisation advantages, these processes optimisation and up-scaling still remain major challenges [40].

#### 2.3.2. Cultures and Lab Grown Proteins

Cultured meat represents a novel biotechnological approach for producing animal proteins without traditional livestock farming. The process typically involves three phases: (a) preparation of cell lines and culture media, (b) cell proliferation and tissue development in bioreactors, and (c) downstream processing [41,42,43].

From an environmental standpoint and when compared with conventional livestock production, cultured meat offers the potential for significant environmental advantages. Life cycle analyses indicate markedly lower greenhouse gas emissions, as well as reduced land and water use, highlighting its potential as a more sustainable alternative to traditional meat [44,45]. Also, from an ethical perspective cultured meat also addresses animal welfare concerns by eliminating the need for large-scale livestock operations. By providing a cruelty-free and environmentally conscious option, it aligns with consumer demands for ethically produced foods [46,47].

Despite its promise, several technological obstacles hinder large-scale commercialisation. These include the development of serum-free media, cost-effective scaffolding systems to support cell growth, and replicating the sensory and nutritional attributes of conventional meat. Current reliance on animal-derived inputs, produced at limited scale, adds further economic and ethical constraints [41,42,48,49].

Other factors that must be considered are the economic implications, as the high cost of culture media remains a critical barrier to scalability and affordability. Beyond cost, consumer acceptance is essential for market success and depends on perceived safety, health benefits, environmental impact, and ethical value. Although surveys suggest growing curiosity and openness toward cultured meat, widespread adoption will require greater transparency, regulatory clarity, and effective communication of its benefits [50].

## 3. Understanding Bioactive Food Proteins

### 3.1. Definition and Significance

Traditionally, dietary proteins have been evaluated primarily for their digestibility and contribution to energy intake. However, this perspective has evolved: proteins are now recognised as modulators of physiological functions that extend beyond basic nutrition. This shift stems from the recognition that certain proteins can actively influence cardiovascular health, metabolic regulation and immune responses, positioning them not only as essential nutrients but also as bioactive agents with tangible health effects [51]. While the concept of bioactive proteins is relatively new in scientific discourse, its roots can be traced back to traditional practices. Fermented foods such as *natto* have long been appreciated in various cultures for their health-promoting properties–understood, at least in part, to be due to the action of bioactive proteins and peptides [52,53].

By definition, a bioactive protein is a naturally occurring protein with the potential to exert specific biological effects, often realised only upon enzymatic cleavage. The active components are typically short peptide (2–20 amino acids) embedded within the primary protein structure and which remain inactive until released through processes such as digestion, food processing, or microbial fermentation [54]. Once freed, these peptides interact with enzymes, receptors, or even transcription factors, modulating a variety of metabolic pathways [55,56].

From a mechanistic standpoint, the biological activity of these peptides is both dose- and sequence-dependent, establishing the pathway for biochemical nutrition [57]. And herein lies the significance of bioactive proteins, as they have not only a great mechanistic diversity by also a wide range of applications. In fact, they have driven the development of value-added foods and enabled tailored strategies in clinical and personalised nutrition [58,59]. Furthermore, their versatility is evident across numerous product categories, ranging from functional beverages and dairy alternatives to sports and medical nutrition formulations. Thus, bioactive proteins represent a bridge between traditional dietary wisdom and modern nutritional science, offering practical health benefits for diverse populations [6,8].

### 3.2. Mechanisms of Bioactivity

Food-derived bioactive proteins and peptides display a remarkable range of physiological activities, mediated by diverse molecular mechanisms. These include enzyme inhibition, modulation of cell signalling pathways, and direct interactions with receptors. Collectively, these actions underpin health-promoting effects such as antihypertensive, anti-inflammatory, antioxidant, antimicrobial, and even opioid-like activities [60].

#### 3.2.1. Receptor Interactions and Signalling Modulation

Many food-derived peptides act as competitive inhibitors of enzymes integral to physiological regulation. A well-established example is the inhibition of angiotensin-converting enzyme (ACE) by short peptides, which results in decreased levels of angiotensin II and induces vasodilation. Tripeptides such as Val–Pro–Pro (VPP) and Ile–Pro–Pro (IPP), released from milk casein during fermentation, exemplify this mechanism. By binding to the ACE active site, these peptides attenuate vasopressor signalling and reduce blood pressure *in vivo* [61,62]. Similarly, several dietary peptides inhibit dipeptidyl peptidase-IV (DPP-IV), an enzyme responsible for degrading incretin hormones. By prolonging the half-life of glucagon-like peptide-1 (GLP-1) and glucose-dependent insulinotropic polypeptide (GIP), DPP-IV inhibitory peptides enhance postprandial glucose regulation. Identified in sources such as egg, milk, and plant proteins, these peptides are of particular interest for managing glycaemia and metabolic health [63].

In addition, numerous peptides inhibit digestive enzymes linked to obesity and metabolic disorders. For instance, peptides derived from plant, marine, and algal proteins can suppress pancreatic lipase, reducing fat absorption and caloric uptake. Such inhibition provides a promising anti-obesogenic strategy to support metabolic health [64].

#### 3.2.2. Enzyme Inhibition

Beyond enzyme inhibition, certain food-derived peptides directly interact with cell-surface receptors or modulate intracellular signalling cascades. A classic example is opioid-like peptides such as β-casomorphins from milk casein and exorphins from wheat gluten, which bind to opioid receptors in the nervous system. By mimicking endogenous opioids, these peptides may exert analgesic or calming effects—for example, whey-derived opioid peptides released during breastfeeding are thought to reduce stress responses in infants [60].

Another example is that peptides influence appetite and satiety through the gut–brain axis. Protein hydrolysates can stimulate satiety hormones such as cholecystokinin and GLP-1 or interact with their receptors, thereby supporting appetite regulation and reducing food intake [60,65].

Another case to be considered lies in the adipose and muscle tissues, where peptides from marine and milk proteins have been shown to activate AMP-activated protein kinase (AMPK), a key regulator of energy metabolism. AMPK activation enhances lipid oxidation and glucose uptake while inhibiting adipogenesis, providing a mechanistic basis for the anti-obesity and antidiabetic potential of these peptides [65,66].

#### 3.2.3. Immunomodulation and Anti-Inflammatory Activity

A substantial subset of food-derived peptides exerts immunomodulatory effects, regulating immune function and attenuating chronic inflammation. Mechanistic investigations showed that these peptides often suppress pro-inflammatory signalling pathways, such as NF-κB, MAPK, and JAK/STAT, thereby reducing the production of cytokines including TNF-α, IL-6, and nitric oxide [67,68]. At the same time, they can enhance anti-inflammatory cytokine expression and antioxidant defences, contributing to a balanced immune response [69].

For example, peptides isolated from fermented dairy and plant products have been shown to inhibit NF-κB activation in vitro, leading to decreased cytokine release and reduced oxidative stress. Some also interact with pattern-recognition receptors on immune cells and gut epithelium, reinforcing mucosal immunity. In vivo studies consistently demonstrate that bioactive peptide mixtures lower pro-inflammatory cytokines (IL-1β, IL-6, TNF-α) and improve clinical outcomes in models of colitis, arthritis, and other inflammatory disorders [70].

#### 3.2.4. Antioxidant and Antimicrobial Mechanisms

Many bioactive peptides display strong antioxidant properties, protecting cells against oxidative stress. Their activity is often linked to specific amino acid residues such as tyrosine, tryptophan, methionine, cysteine, and histidine, which can donate electrons or hydrogen atoms to neutralise free radicals. Legume-, cereal-, and seed-derived peptides have demonstrated potent radical-scavenging activity in vitro and protective effects in cellular and animal models [71]. Beyond direct scavenging, some peptides activate endogenous antioxidant pathways, notably through the Nrf2 signalling cascade, which upregulates antioxidant enzyme expression [71,72].

In parallel, antimicrobial peptides (AMPs) from sources including milk, egg, fish, and insects disrupt microbial membranes or interfere with pathogen metabolism. Their amphipathic, cationic structures allow them to insert into bacterial membranes, inducing cell lysis. Notable examples include lactoferricin (milk-derived) and defensins (insect-derived), both effective against a wide range of pathogens [73,74].

Antioxidant and antimicrobial functions frequently act synergistically—limiting oxidative stress, combating infection, and supporting immune defence—ultimately contributing to improved health [75].

#### 3.2.5. Metabolic Modulation, Anti-Obesogenic Effects, and Gut Microbiota Interaction

Bioactive peptides also contribute to metabolic homeostasis. By downregulating adipogenic transcription factors (PPAR-γ, C/EBPα) and activating catabolic pathways such as AMPK, they inhibit adipogenesis while promoting lipolysis and fatty acid oxidation. In animal models, supplementation with fish- or algae-derived peptides reduced weight gain, improved lipid profiles, and enhanced hepatic fat metabolism. Certain peptides also suppress appetite by modulating gut hormones (GLP-1, PYY) or acting on central receptors, providing a dual strategy of reducing caloric intake and increasing energy expenditure. Other mechanisms include promoting apoptosis of mature adipocytes and regulating metabolic enzyme activity, further underscoring their anti-obesogenic potential [65,66].

An emerging area of research concerns gut–microbiota interactions. Many peptides exert local effects in the gastrointestinal tract, serving as prebiotic-like substrates that stimulate beneficial microbes and short-chain fatty acid (SCFA) production. For instance, egg white hydrolysates increased *Lactobacillus* populations and butyrate levels *in vivo*. Other peptides act as nitrogen sources for microbial growth or directly inhibit pathogenic bacteria, supporting a balanced microbiome. Through these microbiota-mediated pathways, peptides enhance intestinal barrier integrity, modulate local immunity, and reduce dysbiosis-related inflammation. Increasingly, their health effects are recognised as gut-centric, with systemic benefits arising from microbial modulation rather than direct absorption [61,75].

Importantly, these mechanisms are not mutually exclusive: a single peptide may combine ACE-inhibitory, antioxidant, and anti-inflammatory functions. Such multifunctionality often results in synergistic effects that amplify health benefits. Comprehensive validation through *in vitro* assays, animal studies, and human clinical trials remains essential to establish efficacy and guide the incorporation of bioactive peptides into functional foods and nutraceuticals [75,76].

### 3.3. Structure and Its Influence on Bioactivity

Underpinning the diverse mechanisms of bioactive peptides is the fundamental principle that their biological activity is dictated by molecular structure. Several structure–activity relationships have been identified as key determinants of functionality.

#### 3.3.1. Chain Length and Molecular Size

Most bioactive peptides are short sequences of 2–20 amino acids. Their small size increases gastrointestinal stability, enhances interaction with molecular targets, and facilitates cellular uptake. For example, di- and tripeptides often exhibit potent ACE-inhibitory activity by fitting precisely into the enzyme’s catalytic site. In contrast, larger peptides or intact proteins usually lack bioactivity until proteolytic cleavage releases active fragment [70,77].

#### 3.3.2. Amino Acid Composition and Sequence Motifs

The presence of particular residues strongly influences peptide activity and specificity. Hydrophobic amino acids (Val, Leu, Ile, Phe) and Pro often characterise ACE inhibitors, enhancing binding affinity and resistance to degradation. Antioxidant peptides typically contain aromatic or sulphur-containing residues (Tyr, Trp, Met, Cys, His), which efficiently neutralise free radicals [70,75].

Antimicrobial peptides (AMPs) are generally cationic and amphipathic, with motifs rich in Arg and Lys that promote electrostatic attraction to microbial membranes, alongside hydrophobic regions that disrupt lipid bilayers. Similarly, receptor-targeting peptides rely on conserved motifs—for instance, food-derived opioid peptides contain an N-terminal Tyr–X–Phe sequence resembling endogenous enkephalins, enabling high-affinity receptor binding. Immunomodulatory peptides also display characteristic motifs, such as Val–Gly–Val or Gly–Pro–Glu, which have been associated with macrophage activation and anti-inflammatory effects [70,77,78].

#### 3.3.3. Conformation and Structural Stability

Higher-order structures further modulate peptide activity. Cyclic peptides or those adopting β-sheet conformations are often resistant to proteolysis and display enhanced receptor affinity. For example, cyclic peptides from plants and marine organisms can survive gastrointestinal digestion and retain potent ACE-inhibitory or anticancer activities. Structural modifications induced by controlled enzymatic hydrolysis—such as increasing β-turn content while reducing α-helix structures—have been correlated with improved antioxidant and ACE-inhibitory properties [78,79].

Overall, peptide structure—including length, amino acid sequence, and conformation—determines bioactive potential. Even small sequence modifications, such as a single amino acid substitution, can shift activity from antioxidant to antimicrobial, highlighting the exquisite specificity of structure–function relationship.

### 3.4. Bioavailability and Efficacy

The in vivo efficacy of bioactive peptides critically depends on their bioavailability–the proportion of ingested peptide that survives gastrointestinal digestion is absorbed into systemic circulation and remains biological active. Several factors influence this process, including gastrointestinal stability, intestinal absorption, food matrix interactions, delivery strategies, microbiome modulation, and pharmacokinetics.

#### 3.4.1. Stability in the Gastrointestinal Tract

After ingestion, peptides encounter acidic gastric pH and proteolytic enzymes such as pepsin, trypsin, and chymotrypsin. While many are degraded, certain short peptides survive digestion long enough to reach the absorptive epithelium. Proline-rich sequences, unusual peptide bonds, and cyclic structures often confer resistance to proteolysis, whereas linear peptides are typically more labile [75,77].

This explains why *in vitro* activity does not always translate *in vivo*—many peptides lose function upon digestion. To address this, simulated gastrointestinal models (e.g., the INFOGEST protocol) are widely used to predict peptide survival. Strategies to enhance stability include N- or C-terminal modification, cyclisation, or substitution with D-amino acids, although such approaches may reduce the “natural” character of food-derived peptides [75,77,80].

Ultimately, only peptides that endure (fully or partially) the digestive process can exert systemic effects. This limitation suggests that some observed health benefits from high-protein diets or hydrolysates may emerge from minimal direct peptide absorption combined with indirect effects upon gut microbiota modulation, immune system regulation and even metabolic effects [75,77]. The existing data suggests that peptides released during digestion, act as nutraceuticals with relevant biological activities (e.g., antihypertensives, antimicrobial, prebiotic) and will either directly influence cellular processes (e.g., anti-inflammatory activities) or will modulate gut microbiota growth or metabolite production leading to beneficial health effects [81,82]. However, this is still an emerging field as evidence supporting these indirect effect in humans is still weak as many in vitro claims are seldom confirmed in in vivo studies [83].

#### 3.4.2. Intestinal Absorption and Transport

Absorption across the intestinal epithelium is another major barrier. While free amino acids and di-/tripeptides are efficiently transported via PepT1, larger peptides (>3 amino acids) face steep restrictions [84]. Some cross through paracellular diffusion, endocytosis, or transcytosis, particularly if cyclic or structurally constrained.

Peptide physicochemical properties strongly influence absorption:

- Hydrophobic peptides may cross membranes more easily but suffer from poor solubility [85].
- Charge plays a role, with neutral or positively charged peptides favoured [86].
- Specific residues (e.g., Pro, Gly) may reduce degradation or facilitate passage [87].

However, after absorption, peptides face further degradation by intracellular peptidases and hepatic first-pass metabolism, greatly limiting systemic bioavailability. Still, numerous short peptides (2–9 amino acids) from soy, corn, lactoferrin, ovotransferrin, and fish proteins have been detected in plasma, demonstrating that intact absorption is possible [75,77].

#### 3.4.3. Effects of the Food Matrix

The food matrix—the context in which peptides are consumed—significantly affects bioavailability. When ingested as part of complex meals, peptide release is slower and absorption may be reduced compared with isolated supplements. For example, a casein-derived tripeptide appeared more slowly and at lower levels in plasma when consumed in yoghurt compared with water [85].

Matrix components also modulate peptide fate:

- Minerals and sugars may enhance proteolysis, reducing availability [88].
- Fats and emulsifiers can protect peptides by encapsulation [89].
- Dietary fibres may restrict enzyme access [90].
- Polyphenols may reduce transporter expression, whereas co-ingested amino acids may upregulate it [91].

Thus, the overall impact of the food matrix can be either protective or inhibitory. Understanding these interactions is critical for designing functional foods that deliver peptides effectively [77].

## 4. Technological Influence on Bioactive Potential

Food processing plays a crucial role in shaping the structural, functional, and physiological properties of proteins. Both conventional and emerging technologies can alter protein conformation, digestibility, and peptide release, thereby modulating bioactivity [92].

### 4.1. Traditional Processing Techniques

Conventional methods such as heating, drying, fermentation, and enzymatic hydrolysis have long been employed to ensure food safety, extend shelf life, and improve sensory qualities [93]. Increasingly, these approaches are also recognised for their impact on bioactive peptide formation:

- Fermentation enhances protein digestibility and promotes the release of bioactive peptides with antioxidant, antihypertensive, and antimicrobial activities. Traditional foods such as natto are rich in nattokinase, a fibrinolytic enzyme with cardiovascular benefits [94]. Similarly, cheese maturation generates opioid peptides and ACE inhibitors through proteolytic activity [95].
- Thermal processing can increase bioactivity by denaturing proteins and exposing cleavage sites for enzymatic hydrolysis. However, excessive heat may induce Maillard reactions that mask bioactive regions or produce undesirable compounds [96,97]. Careful optimisation is therefore needed to maximise health-promoting properties while minimising negative effects.
- Enzymatic hydrolysis remains one of the most effective strategies for generating bioactive peptides in a targeted manner, as demonstrated by whey hydrolysates and fermented legumes [98].

### 4.2. Modern Processing Techniques

Emerging non-thermal and combined approaches are being investigated to improve the yield, stability, and bioactivity of food-derived peptides:

- High-pressure processing (HPP) induces conformational changes in proteins, enhancing their susceptibility to hydrolysis and peptide release [99,100].
- Pulsed electric fields (PEF) disrupt cell membranes, improving enzyme accessibility and extraction of bioactive compounds [101].
- Ultrasound-assisted extraction generates acoustic cavitation, which promotes efficient protein hydrolysis and has been shown to increase antioxidant and antihypertensive activity in soy and dairy proteins [102].
- Cold plasma is under exploration for its ability to modify protein structures without extensive thermal damage, potentially enhancing bioactivity [103,104].

Synergistic applications—such as combining HPP with enzymatic hydrolysis or fermentation—are particularly promising, as they maximise both peptide yield and functional quality [105,106]. This trend toward precision processing aims to tailor specific peptide profiles to desired health outcomes, paving the way for the design of targeted functional ingredients.

A comparison between examples of classical and modern techniques can be found in Table 1.

## 5. Applications in Food and Nutrition

The integration of bioactive proteins into food and nutrition systems provides a dual opportunity: delivering high-quality nutrients while offering specific physiological benefits. Sources include plants, fish and marine by-products, insects, microalgae, fungi, and agri-food residues [118,119]. Protein hydrolysates and peptides obtained through enzymatic or microbial processes have been shown to exert antioxidant, antihypertensive, anti-inflammatory, immunomodulatory, and antidiabetic effects [120,121]. Their applications span functional foods, nutraceuticals, and clinical nutrition.

### 5.1. Functional Foods and Beverages

Functional foods and beverages are formulated to provide health benefits beyond basic nutrition. In this context, bioactive proteins serve both as macronutrients and as carriers of bioactivity [122]:

- Antihypertensive peptides, especially ACE inhibitors derived from marine, dairy, or legume proteins, can be incorporated into yoghurts, dairy-free drinks, and ready-to-eat meals, where they help regulate blood pressure [123,124,125].
- Antioxidant peptides reduce oxidative stress by neutralising reactive oxygen species and activating redox-sensitive pathways such as Nrf2/ARE, with potential roles in disease prevention [126,127].
- Satiety-inducing peptides stimulate gut hormones like GLP-1 and PYY, supporting appetite control and weight management. For example, pea and insect protein hydrolysates have been associated with increased satiety signalling [128,129,130,131].

Formulation challenges include the bitterness of hydrolysates, stability in complex matrices, and retention of activity during processing or storage. Encapsulation technologies (e.g., spray-drying, liposomes, protein–polysaccharide complexes) are increasingly used to mask off-flavours, protect peptides, and improve delivery [132,133,134,135].

### 5.2. Nutraceutical and Dietary Supplements

Nutraceuticals offer a convenient format for delivering concentrated bioactive peptides outside of complex food systems [136,137], and they typically target key health domains such as immune support, musculoskeletal health, cardiovascular regulation, and metabolic function [138]:

- Anti-inflammatory peptides from fish skin, soy, lupin, or insects downregulate cytokines (IL-6, TNF-α) and inhibit NF-κB signalling, making them relevant for individuals with low-grade inflammation or metabolic syndrome [139,140,141].
- Collagen peptides support joint, bone, and skin health, with growing interest in marine and insect-derived alternatives to bovine or porcine collagen [142].
- Sports nutrition products use protein hydrolysates enriched in branched-chain amino acids (BCAAs) to stimulate muscle protein synthesis and recovery [143]. Fast-absorbing hydrolysates from whey, soy, and insect larvae (*Hermetia illucens*) are being used for pre- and post-exercise supplementation [144,145].

To enhance bio-efficacy, nano- and microencapsulation systems (e.g., protein–lipid carriers, biopolymer matrices) are employed to improve peptide stability against gastrointestinal degradation and to achieve targeted release [146]. The valorisation of food industry by-products as raw materials for peptide production also aligns with circular bioeconomy goals, reducing food waste while adding value to supplement formulations [147].

### 5.3. Medical and Clinical Nutrition

In clinical settings, bioactive proteins are incorporated into specialised formulations for patients with malnutrition, chronic disease, or compromised gastrointestinal function [148]:

- Protein hydrolysates are favoured for their digestibility, low allergenicity, and improved absorption. Hypoallergenic formulations from rice or pea proteins are especially valuable for patients with food sensitivities [149,150,151].
- Anabolic peptides stimulate muscle protein synthesis via mTOR and Akt signalling, helping counteract sarcopenia in elderly or immobilised patients [152,153].
- Immunomodulatory peptides from casein, soy, or marine proteins support immune function in patients with chronic inflammation or immune dysregulation [154].
- Gut-targeting peptides modulate microbiota composition and enhance barrier function, with potential benefits for inflammatory bowel diseases and recovery after antibiotic treatment [155,156,157]

Novel sources such as insects and algae are being explored for clinical nutrition, although regulatory approval and consumer acceptance remain limiting factors [158,159]. From a sustainability perspective, upcycled by-products (e.g., fish frames, defatted insect meal, microalgal biomass) could lower costs and enhance the environmental efficiency of clinical nutrition products, particularly in public health systems [160,161]

## 6. Current Outlook and Perspectives: Market, Challenges and Limitations

The global market for protein ingredients is projected to expand significantly in the coming years, driven by population growth, health awareness, and sustainability concerns. Consumers increasingly associate protein with satiety, weight management, and disease prevention, fuelling demand for both traditional and alternative sources. However, the transition toward bioactive and novel proteins is shaped by multiple opportunities and barriers across economic, technological, and social dimensions.

The industry still faces a number of obstacles, such as unclear regulations, challenges with large-scale production, gaining public trust, and navigating intellectual property rights.

### 6.1. Intellectual Property (WIPO–Patent Scope)

An analysis was conducted in the WIPO (World Intellectual Property Organisation) database using the PATENTSCOPE search tool (https://www.wipo.int/en/web/patentscope), considering patents published in the last 15 years (2010–2025) using the keywords “Alternative protein” and “Food” (FP: Alternative protein AND Food, accessed on 18 July 2025). The filters implemented were: (i) Language English; (ii) Stemming activated (search according to the stem or root form), (iii) One Single Family Member, (iv) Not include Non-patent literature.

**Figure 1:**
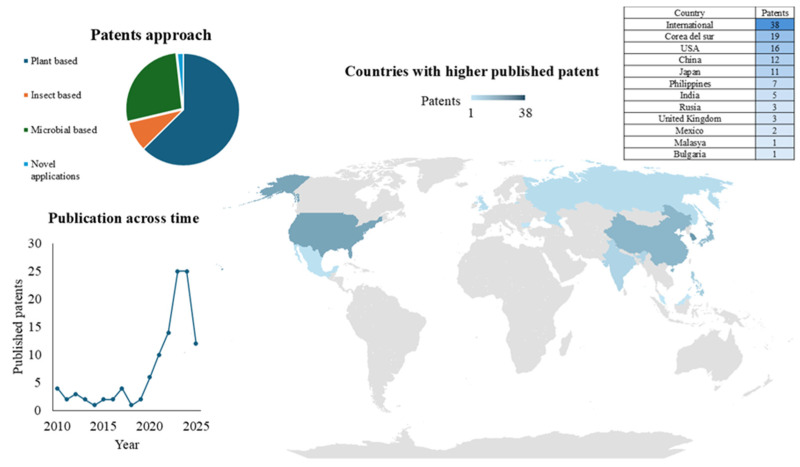
Temporal, geographical, and technological distribution of published patents on alternative protein sources (WIPO-PATENTSCOPE, accessed on 18 July 2025).

The results enabled the recovery of 190 patent records that utilise alternative protein as a primary component. A subsequent filtering of duplicate patents or those focused outside the food sector was performed. This allowed the recovery of 115 intellectual property records (Figure 1).

An analysis of the number of publications over the years reveals that, until 2020, fewer than six patents were published annually. After 2021, the minimum number of patents was 10, indicating that the years with the highest number of publications are 2023 and 2024. This growth may be related to the continuous promotion by the “World Health Organization, WHO” to find solutions to replace animal-derived proteins with sustainable alternatives, as well as the interest of experts (scientists and nutritionists) in new sources (plants, insects, microalgae, among others) [162].

Additionally, the patents (115) can be classified into three groups according to the protein source involved: (1) Plant-based protein (72 patents), (2) Insect-based protein (10 patents), and (3) Microorganism-based protein (31 patents). Likewise, a 4th group was added according to novel applications in the market (2 patents). Thus, the most significant number of registered patents are under the use of plant-based proteins; this makes sense given the greater integration of vegan products in the market and the normality of implementing plant-based products in the diet; Siddiqui, et al. [43] point out that “Food familiarity is also a significant contributor to food motivation”, possibly familiarity with plant-based products in other aspects of the diet promotes more consumer interest in consuming plant-based proteins; the opposite is the case with microorganisms and specifically insects. The behaviour previously described by Otero, et al. [163] highlights the low percentage (3.3%) of patents related to insects as a source of protein, which they attribute to the initial stages of interest in this new source of protein. For its part, in the integration of new technologies, two patents (US 419726481 and WO 2022204122) are highlighted, focusing on the application of “Machine learning” for the selection and identification of natural ingredients. This is evidence of how new trends are also promoting the integration of alternative proteins in the market. Finally, the countries with the highest publication of patents related to alternative proteins are: Republic of Korea (19), USA (16 patents), China (12) and Japan (11), the above agrees with what was described by the study by Lee, et al. [164], where they point out that investment, technological development and consumption are oriented by North America and Europe, highlighting that the protein analogues market (Meat) is mainly focused on North America, Europe and Asia-Pacific; therefore, the fact that countries in these regions lead the number of registered patents would corroborate the development of technological interest. Examples of applications of these patents can be found in Table 2.

### 6.2. Trends Analysis (VOS Viewer)

In addition to analysing published patents, we also examined the literature published in recent years (2020–2026), comprising a total of 4368 research articles registered in the Scopus database (accessed on 24 July 2025), using the keywords “Alternative protein” and “Food product.” Subsequently, a co-occurrence analysis was performed by creating a thesaurus to eliminate repetitions and synonyms (e.g., proteins/protein; plants/plants; fungal/fungi).

**Figure 2:**
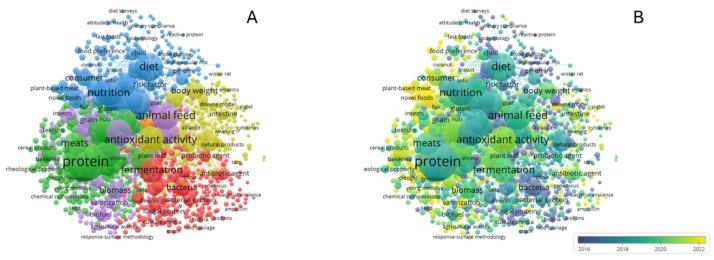
Network graph of alternative protein research from 2020 to 2026 by VOSviewer version 1.6.20 (Scopus database, accessed 24 July 2025). (**A**) Cluster distribution of research information in alternative protein research; (**B**) Time distribution between 2020 and 2026.

The analysis allowed the separation of the information into five clusters (Figure 2A):

(1) Fermentation and microbiology approach (Red): Studies focused on the production of microorganisms through fermentation (fungi, bacteria, yeasts, microalgae, etc.) for the production of specific proteins (bacteriocins and enzymes). Additionally, some studies focused on heterologous production—keywords: Fermentation, bacteria, food preservation, and recombinant protein.
(2) Protein in the food industry (green): Studies focused on the application of protein in food (meats), as well as studies focused on animal protein (whey) and plant protein (soybean protein); as well as sensory studies, process analysis, and applications in the food industry in sectors such as bakeries and meat. Keywords: Protein, plant protein, food industry, meat, texture analysis, bakery, and food production.
(3) Alternative protein, nutritional and consumer approach (Blue): Studies focused on the elucidation of new alternative protein sources (insects, microalgae) and the development of new analogous products (meat). Likewise, some topics of interest in this cluster include aspects of nutritional value, health, diet, and risk factors. Finally, some consumer aspects, such as preference, acceptability, and food neophobia, have been included here—keywords: nutrition, Alternative proteins, diet, and nutritional value.
(4) Health benefits and dietary supplements (Yellow): Studies focusing on the positive health benefits (antioxidant, anti-inflammatory) of dietary supplements, animal model studies, and their impact on the microbiota are visualised in this cluster—keywords: Antioxidant activity, body weight, dietary supplement, intestine, and drug effect.
(5) Environmental and valorization approach (Violet): Studies that focus on new trends in the circular economy through the integration of by-products to generate value-added products. Keywords: Biomass, microalgae, biofuel, food, and animal feed.

Additionally, an analysis of the data over time (Figure 2B) indicates that the recently published area (yellow, 2021–2025) encompasses clusters 2, 3, and 4, which relate to the application of proteins in the food sector, the search for alternatives and the valorization of by-products. Thus, the data follows the growth already described in the patent analysis section, in line with the growth caused by the continuous promotion of the search for sustainable alternative proteins [162].

### 6.3. Challenges and Limitations (Legislation)

While the excitement around bioactive proteins is growing, several important challenges still stand in the way of their widespread adoption.

#### 6.3.1. Regulatory Uncertainty

One of the biggest hurdles is the lack of clear, consistent regulations across different countries. Many regulatory systems have yet to develop harmonised guidelines for evaluating bioactive peptides, especially those derived from less traditional sources like insects, mycoproteins (fungus-based proteins), or compounds generated through fermentation processes [166,167]. For example, the approval of insect-based protein products varies widely—while some countries in the EU are pioneering regulations for edible insects, others lag behind, slowing market entry [168]. Similarly, mycoprotein products like Quorn have faced extensive regulatory scrutiny to prove safety before gaining acceptance in different regions [169]. This patchwork of regulations creates uncertainty for companies and slows down the commercialization of innovative bioactive protein products.

#### 6.3.2. Ensuring Safety

Safety is paramount when introducing new proteins into the food supply. Novel bioactive proteins require thorough risk assessments to rule out potential allergenic reactions, ensure they are free from harmful microorganisms, and confirm they can be properly digested and metabolised by humans [167,170]. For instance, fermentation-derived proteins sometimes carry concerns about residual microorganisms or toxins, which demand rigorous quality control [171]. Clinical trials like those conducted on pea protein isolates or cultured meat prototypes have been essential in demonstrating digestibility and safety before wider release [172]. These assessments are often time-consuming and costly but crucial for consumer protection.

#### 6.3.3. Intellectual Property Complexities

Protecting innovations in bioactive proteins can be tricky. The broad scope of bioactive claims and repetitive nature of peptide sequences pose challenges for securing patents. For example, companies working on novel peptides derived from fermentation or insects face obstacles in patenting sequences that might be naturally occurring or previously documented [173,174]. Furthermore, bioprospecting in traditional communities raises questions about how to honour indigenous knowledge fairly. Initiatives like the Nagoya Protocol aim to ensure benefit-sharing when genetic resources are used commercially, but navigating these agreements can slow innovation [175]. Some companies pursue trade secrets or proprietary processing methods to protect their investments where patents are difficult.

#### 6.3.4. Consumer Acceptance

Last but not least, when considering alternative proteins, one has to consider the true final hurdle: consumer acceptance. This is greatly influenced by various factors such as familiarity, food neophobia (fear or reluctance to try new and unfamiliar foods), disgust, and cultural norms [43]. Among the main challenges associated with this topic are the lower willingness of consumers to accept alternative proteins sources, the products novelty and concerns about product quality, especially when comparing these proteins with those of animal origin [176,177]. Additionally, there is a dissonance between stakeholders and the consumer’s, as the first are aware of the sustainability implications of alternative proteins [178].

One way to bypass these limitations is through consumer education and awareness on the topic. This can be achieved through educational interventions, persuasion, training, and modelling approaches on the advantages, disadvantages and benefits of alternative proteins [43]. As studies have shown that social norms, more than individual drivers and perceptions, are highly relevant in accepting alternative proteins, marketing plays have been single out as being one of the most viable strategies to increase alternative proteins acceptance [179,180]. Investing in productizing, packaging and branding is being touted as one key drivers in consumer education, with data analysis showing that providing consumers with a sense of familiarity and comfort is key to their acceptance and uptake of alternative proteins [181].

## 7. Conclusions

Proteins are essential macronutrients, but their relevance extends far beyond basic nutrition. Bioactive proteins and peptides play pivotal roles in regulating blood pressure, modulating immunity, reducing oxidative stress, and supporting metabolic health. Advances in food technology and biotechnology have broadened the range of protein sources—from plants, insects, and algae to microbial and cultured proteins—while also enabling more precise processing strategies to enhance bioactivity. This innovation will likely be driven by precision processing, sustainable sourcing, and personalised nutrition applications. Valorisation of agri-food by-products, combined with novel biotechnological approaches, offers an opportunity to create cost-effective and environmentally responsible protein ingredients.

However, despite these advances, challenges and hurdles remain. Ensuring the bioavailability and stability of peptides, overcoming sensory and formulation barriers, reducing production costs, and addressing regulatory complexity are all critical for the successful integration of bioactive proteins into mainstream food systems. Consumer acceptance is equally decisive, influenced by perceptions of safety, sustainability, and ethical production.

Nevertheless, bioactive proteins future is bright and is a promising avenue in the development of sustainable and functional nutrition.

## Figures and Tables

**Table 1:** Impacts of processing techniques on bioactivity of food proteins.

Technique	Description	Effect	Examples	References
Thermal processing	Heat-based treatment	Denaturing, aggregation or Maillard reactions significant alter proteins and leads to enhanced or reduced bioactivity	Pasteurised milk proteins, cooked soy	[93,107,108]
Enzymatic hydrolysis	Enzyme mediated cleavage	Protein breakdown into bioactive peptides enhances bioactivity and reduces allergenicity	Whey hydrolysates, fermented legumes	[109,110]
Fermentation	Microbial conversion	Enhanced peptide content and diversity with diverse bioactivity profile and enhanced digestibility	Natto, kefir, fermented cereals	[106]
High-Pressure Processing	>400 MPa pressure application	Increased peptide accessibility due to protein structural changes. Enhanced digestibility while maintain bioactivity	Meat analogues, dairy alternatives	[111,112]
Ultrasound-assisted extraction	Acoustic cavitation	Cavitation effect disrupts cell walls and boosts peptide yield without deleterious effects for bioactivity	Pea and soy protein isolates	[113,114]
Pulsed Electric Field	Electric pulses to disrupt cells	Induces protein unfolding and aggregation without deleterious effects, enhancing bioactive compounds release and digestibility	Fermented beverages, plant protein mixes	[115,116,117]

**Table 2:** Recent application of alternative-based protein in food products.

Origin	Species	Food Application	Formulation	Relevant Results	Reference
Insect	*Alphitobius diaperinus* (*Whole Buffalo Powder*)	Soy-protein-based burgers	Burgers 5% (B5) and 10% (B10) insect protein	No significant effect of the addition of insect protein on the cooking efficiency and the parameters of texture profile analysis. Increasing total lipids and saturated fatty acids (SFA) and decreasing monounsaturated (MUFA) and polyunsaturated fatty acids (PUFA).	[29]
	*Mealworm*, *Migratory locust*, *House cricket*	Meat extenders in beef burgers	5% (*w*/*w*) of each insect powder	The formulation (5%, mealworm burgers) showed higher sensory acceptability. Cricket formulations reduced the accumulation of heterocyclic amines (potential carcinogenic molecules) after cooking. Potential modulation of pancreatic lipases (through interactions with bioactive peptides) slows lipid digestion.	[28]
Mycoprotein	*Fusarium venenatum*	Mycoprotein-Based Harbin Red Sausages	Lean pork meat with *F. venenatum* (0, 25, 50, 75, 100%)	In terms of redness and thawed water component, there was no significant difference between the red sausage (25%) and the control. The flavour substances were slightly richer, and the consumer preference was higher.	[35]
	*Aspergillus oryzae*	Burger patties	Mycoprotein 55%	The product provided more than 20% of the protein required (reference nutritional values). The shelf-life analysis revealed that the physical characteristics remained unchanged over a 14-day interval.	[36]
Microalgae	*Nannochloropsis oceanica*	Plant-based fishcake analogue	*N. oceanica* (0, 10, 20, and 30%)	Changes in the physical properties of PFC (increased hardness, decreased springiness, reduced moisture and oil contents, and stronger gel strength). *N. oceanica* in PFCs led to a suppression in the release of specific amino acids and fatty acids, which could potentially impact amino acid and fatty acid metabolisms. The addition of *N. oceanica* concentration (10%) in PFC increased the in vitro protein digestibility.	[165]
	*Spirulina platensis*	Protein-based emulsion gel as a fat substitute	SPP: *S. platensis* protein nanoparticles (1%, *w*/*v*) and soybean oil (SO) (Ratio 50:50). SPP-XG: SPNPs-based pre-emulsions mixed with 2% xanthan gum (XG) solution in a ratio of 8:2	The addition of SP-analogues yielded similar data to conventional fat analogues in terms of shrinkage (10.30–18.71%) and cooking losses (17.23–29.58%). An increased amount of free water (pullulan proteins) was detected in the boiled and steamed cooking process, which could enhance water mobility and initial succulence.	[122]

## Data Availability

Not applicable.
